# Development and Characterization of Active Gelatin Films Loaded with Rapeseed Meal Extracts

**DOI:** 10.3390/ma14112869

**Published:** 2021-05-27

**Authors:** Alicja Tymczewska, Bliss Ursula Furtado, Jacek Nowaczyk, Katarzyna Hrynkiewicz, Aleksandra Szydłowska-Czerniak

**Affiliations:** 1 Department of Analytical Chemistry and Applied Spectroscopy, Faculty of Chemistry, Nicolaus Copernicus University in Toruń, Gagarina 7, 87-100 Toruń, Poland; 285474@stud.umk.pl; 2 Department of Microbiology, Faculty of Biological and Veterinary Sciences, Nicolaus Copernicus University in Toruń, Lwowska 1, 87-100 Toruń, Poland; bliss.furtado@umk.pl (B.U.F.); hrynk@umk.pl (K.H.); 3 Department of Physical Chemistry and Physicochemistry of Polymers, Faculty of Chemistry, Nicolaus Copernicus University in Toruń, Gagarina 7, 87-100 Toruń, Poland; janowa@umk.pl

**Keywords:** active packaging, gelatin films, rapeseed meal extracts, mechanical properties, morphology, radical scavenging activity, antibacterial activity

## Abstract

The use of industrial waste as a material for the development of natural innovative and active packaging is economically and environmentally appealing. The aim of this study was to develop and characterize active gelatin films incorporating rapeseed oil industry waste. Water (RM-WE) and methanolic (RM-MWE) extracts of rapeseed meal (RM) were used as active agents in film formulations. The active films were produced by a casting technique. The physicochemical, mechanical, optical, morphological, radical scavenging, and antibacterial properties of the films were analyzed. The addition of RM-WE and RM-MWE in the concentrations range between 4 and 12% promoted an increase of Young’s modulus (YM) and radical scavenging properties of films investigated by the direct QUick, Easy, New, CHEap and Reproducible procedure using 2,2-diphenyl-1-picrylhydrazyl (QUENCHER_DPPH_) and 2,2′-azino-bis(3-ethylbenzothiazoline-6-sulfonic acid) (QUENCHER_ABTS_) radicals. The antibacterial properties of films were examined against five bacterial strains: *E. coli*, *S. enterica*, *M. luteus*, *L. monocytogenes*, and *S. aureus*. Additionally, color and opacity of the control and fortified films differed significantly. The gelatin films with RM extracts are resistant to the microbial spoilage and could be used to produce active packaging for food that is vulnerable to rancidity effects.

## 1. Introduction

Recently, there has been a substantial interest in the use of naturally occurring polymers, such as proteins for preparing eco-friendly and safe films, which generate considerable attention in the food industry. These films are becoming popular food packaging materials not only because of their biodegradability, but also due to the possibility of films’ improvement by combining or blending with substances possessing antioxidant and/or antimicrobial properties [1,2].

One of the most extensively studied protein-based material, mostly due to its ability to protect food against light, oxygen and drying, is gelatin [3]. However, the main problems with application of the gelatin-based films are their poor water resistance, low tensile strength, and vulnerability to microbial degradation. In order to improve gelatin properties, it is necessary to supplement the polymer with functional additives, such as plant-based natural extracts [4,5,6,7]. Currently, researchers are focused on exploring natural plant extracts for application as functional additives in innovative packaging materials. The use of natural plant extracts is an attractive alternative to synthetic additives since they possess antibacterial compounds that can control the presence of foodborne pathogens on one hand and can safely contact with food. Therefore, some plant extracts can improve the packaging efficiency and extend the shelf life of food products [8]. Nowadays, one can observe increasing demand for natural and safe food packaging, as well as pressure for replacing synthetic chemical compounds due to their potential health risks both by the consumer and the food industry. Furthermore, there is a feasibility to develop sustainable and low-cost coatings simultaneously.

Processes such as oil, juice or sugar production significantly contribute to the formation of enormous amounts of waste. These kinds of residues are widely used as a feed or compost, but, in fact, by-products of the food industry can be an attractive sources of bioactive compounds, which enhance the desirable properties of packaging. Moreover, recovering substances that can be incorporated in coatings, promote the “zero waste” movement [9,10]. There are several examples of active agents that can be extracted from agro-food waste, such as fruit and vegetable peels or oil industry by-products, which when mixed with biopolymer matrix provide promising results [4,11,12,13,14].

Rapeseed (*Brassica napus* L.), as a crop, is widely cultivated worldwide and especially in Continental Europe (Germany, France and Poland), China, India, and Canada. Processing of rapeseed generates tens of millions of tons of side-stream materials due to technological conditions of edible oil production [15]. Rapeseed meal (RM) is a by-product of rapeseed oil extraction and, due to a high protein content (34–45%), it is still widely applicable as an addition to livestock feed. The fibers and undesirable anti-nutritional compounds, such as glucosinolates, phytates, and isothiocyanates, present in RM generate limitations in its utilization as animal feed [16,17]. Therefore, anti-nutrient removal is crucial for allowing RM to be applied as a food additive or dietary supplement with possible health-promoting properties. On the other hand, RM has notable amounts of phenolic compounds, mainly sinapic acid and its derivatives: sinapine, 4-vinylsyringol and tannins with high antioxidant potential [18]. Hence, RM after removal of anti-nutrients can be used in human diet or in food preservation and packaging. The rapeseed protein isolate and rapeseed powder originating from non-genetically modified double low (00) *Brassicaceae* species with a low content of erucic acid and reduced content of glucosinolates were judged as safe ingredients in human food products by the European Food Safety Authority (EFSA) [19,20]. Moreover, rapeseed by-products containing strong antioxidants had the ability to protect DNA from damage in the presence of a radical inducer and inhibited acetylcholine esterase (AChE) enzyme activity. Interestingly, sinapine is a viable AChE inhibitor for neurodegenerative and muscle diseases [21,22]. Additionally, bioactive compounds of the *Brassicaceae* family exhibited varying antimicrobial (antibacterial and antifungal) activity. Engels et al. [23] showed that the crude extract of oriental mustard (*Brassica juncea* L.) seed meal and purified polyphenols had selective antibacterial effects on Gram-positive bacteria (*Listeria monocytogenes* and *Staphylococcus aureus*) and Gram-negative bacteria (*Escherichia coli*, *Bacillus subtilis*, and *Pseudomonas fluorescens*). Another study, by Miceli et al. [24], reported the antibacterial activity of the aqueous extracts obtained from the leaves of *Borago officinalis* L. and *Brassica juncea* L. The antagonistic activity was evaluated against several bacteria (42 strains of *Listeria monocytogenes*, 35 strains of *Staphylococcus aureus*, 38 strains of *Enterobacter* spp. and 18 strains of *Salmonella enterica*) commonly associated with foodborne diseases.

Therefore, the future direction of research could focus on using RM as a source of active substances for the synthesis of innovative packaging materials. Recently, biopolymer films based on RM protein with various modifications (e.g., cross-linking, incorporating chitosan, cellulose fiber, polycaprolactone) have been successfully formulated and their mechanical, optical, thermal, and antibacterial properties have been examined [25,26,27,28].

However, to the best of our knowledge, there has been no reference on studying the changes in physico-mechanical, optical, morphological, antioxidant, and antimicrobial properties of gelatin-based films spiked with different concentrations of RM extracts.

In this context, the objective of the present work was to evaluate the effects of different concentrations of water (RM-WE) and methanolic (RM-MWE) RM extracts on selected physicochemical, mechanical, optical, morphological, radical scavenging and antibacterial characteristics of gelatin films for active packaging application. The potential antibacterial activities of gelatin films incorporating RM-WE and RM-MWE against major foodborne pathogens, namely, two Gram-negative bacteria: *Escherichia coli*, *Salmonella enterica*, and three Gram-positive bacteria: *Micrococcus luteus*, *Listeria monocytogenes*, and *Staphylococcus aureus*, which are responsible for many health-related problems, were studied. Moreover, principal component analysis (PCA) was applied to classify and discriminate samples of gelatin-based films loaded with different concentrations of RM-WE and RM-MWE.

## 2. Materials and Methods

### 2.1. Chemicals and Materials

All chemicals used in the study were of analytical or HPLC grade. Gelatin from bovine skin (20 mesh) was purchased from Chemland (Stargard Szczeciński, Poland). Rapeseed meal (RM) in the original packaging (polyethylene film) was kindly donated by the local vegetable oil factory. RM is a by-product obtained from the dehulled and flaked rapeseed by extracting oil with hexane and then desolventizing the defatted flakes by means of high temperature thermal processing.

### 2.2. Preparation of Rapeseed Meal Extracts

In this study, distilled water and methanol–water (1:1 *v*/*v*) were used for extraction of antioxidants from RM. A 2.0 g portion of ground RM and 20 mL of each solvent were transferred into round-bottomed flasks and shaken using a shaker SK-L 330-Pro (Chemland, Stargard Szczeciński, Poland) at room temperature for 30 min. Each sample was extracted in triplicate, and the residual rapeseed flour was separated by centrifugation (centrifuge MPW-54, Chemland, Stargard Szczeciński, Poland, 4500 rpm, 10 min). The pooled extracts were filtered and stored in a refrigerator prior to the analysis.

### 2.3. Preparation of Active Films

The filmogenic solutions were prepared by mixing gelatin (polymer matrix, 5% *w*/*w* in each film), glucose and glycerol (plasticizers, 1.25% *w*/*w* in each film), RM extract (antioxidant agent, 4, 8, and 12% of RM-WE and RM-MWE, respectively) with distilled water. The resulting solutions were mixed with a magnetic stirrer (RH Basic 2, IKAPOL, Warszawa, Poland) at elevated temperature 333 K for 15 min. Then, the mixtures were sonicated for 2 min using an ultrasonic clearer bath (5200DTD, Chemland, Stargard Szczeciński, Poland) to remove air bubbles. The film-forming solutions were poured into Petri dishes and left to dry at room temperature for 48 h. After drying, the films were peeled off from the casting surface.

### 2.4. Physicochemical, Optical and Morphological Properties of Films

#### 2.4.1. Moisture Content

The moisture content (MC) in the obtained films was determined by the gravimetric method. A square (1 cm × 1 cm) sample was cut from each film and weighed (W_i_–the initial weight). Then, squares were dried at 105 °C for 3 h in a drying oven (SUP-3, Zalmed, Warszawa, Poland) and weighed again to determine the final dry weight (W_f_). The moisture content in each film was analyzed in triplicate and calculated using Equation (1):

(1) $$\mathrm{MC}\;\left(\%\right)=\frac{W_{i}-W_{f}}{W_{i}}\times 100$$

#### 2.4.2. Mechanical Properties

Mechanical properties such as the modulus of elasticity—Young’s modulus (YM), tensile strength (TS) and elongation at break (EAB) of studied films were measured according to modified ISO 527-3:2018 standard [29] using the universal testing machine Shimadzu EZ-test SX (Kyoto, Japan). The film samples in a form of a uniform strap (50 mm × 10 mm and known thickness) were placed between metal clamps (with 30 mm distance between them). The samples were stretched with a constant speed of 10 mm/min. The TS and EAB were determined directly from the plotted stress–strain curves, the YM was estimated from the slope of the elastic region (initial linear portion of the plot). Each measurement was repeated three times.

#### 2.4.3. Surface Color and Opacity Measurement

Color parameters in the CIE L* a* b* system were determined using a MICRO-COLOR II LCM 6 spectrophotometer (Dr. Bruno Lange GmbH & Co. KG, Berlin, Germany). Within the CIE-Lab system, the color is defined using three parameters: lightness (L*) and chromaticity parameters redness (a*) and yellowness (b*) of the specimen. The measurements were conducted in five replications and the total color difference value (ΔE) was calculated using Equation (2):

(2) $$\Delta E=\sqrt{\left[{\left({\Delta L}^{*}\right)}^{2}+{\left({\Delta a}^{*}\right)}^{2}+{\left({\Delta b}^{*}\right)}^{2}\right]}$$

where ${\Delta L}^{*}$, ${\Delta a}^{*}$, ${\Delta b}^{*}$ refer to the differences between the color value parameters of control film and gelatin films incorporated with different concentrations of RM-WE and RM-MWE.

Opacity of the obtained films was measured according to the method proposed by Wang et al. [30]. Each film was cut into a rectangular piece and placed in the test cell of a Hitachi U-2900 spectrophotometer (Tokyo, Japan). Then, the absorbance at 600 nm was measured five times. Film opacity was calculated by Equation (3):

(3) $$\mathrm{Opacity}=\frac{{\mathrm{Abs}}_{600}}{x}\;\left({\mathrm{mm}}^{-1}\right)$$

where ${\mathrm{Abs}}_{600}$ is the value of absorbance at 600 nm and x is the film thickness (mm).

#### 2.4.4. Scanning Electron Microscopy

Scanning electron microscopy (SEM) cross-section imaging was performed using the LEO1430 VP machine (Leo Electron Microscopy Ltd., Cambridge, UK). Broken samples examined in mechanical tests were sputtered with thin layer of gold to improve layer conductivity.

### 2.5. Radical Scavenging Activity of Rapeseed Meal Extracts and Films

In present study, the modified 2,2-diphenyl-1-picrylhydrazyl (DPPH) and 2,2′-azino-bis(3-ethylbenzothiazoline-6-sulfonic acid) (ABTS) assays previously described in detail [31] were used for the evaluation of radical scavenging activity (RSA) of the RM-WE and RM-MWE, while the QUick, Easy, New, CHEap and Reproducible (QUENCHER) procedure was applied for direct determination of radical scavenging properties of the solid film samples.

According to QUENCHER_DPPH_ procedure, film samples (0.1 g) were ground in an electric laboratory mill (FW100, Chemland, Stargard Szczeciński, Poland). Then, 6 mL of DPPH solution was added to test tube containing the sample. In the next step, samples were shaken vigorously (Classic Vortex Mixer, Velp Scientifica Srl, Usmate (MB), Italy) for 10 min to facilitate the reaction with the reagent. After shaking, test tubes were put in the dark for 15 min. After this time the absorbance of the optically clear supernatant was measured spectrophotometrically at 517 nm using Hitachi U-2900 spectrophotometer (Tokyo, Japan).

According to QUENCHER_ABTS_ procedure, 6 mL of ABTS radical cation solution diluted with ethanol to an absorbance of 0.70 ± 0.02 at 734 nm and weighed film sample (0.1 g) were shaken vigorously for 10 min to facilitate the reaction with the reagent. After 1 min of incubation at 40 °C, the absorbance of the optically clear supernatant was measured spectrophotometrically at 734 nm.

The RSA analyses were performed in five replications and the results were expressed as μmol Trolox equivalents (TE) per 100 g of sample.

### 2.6. Antibacterial Activity of Films

#### 2.6.1. Bacterial Strains and Inoculum Preparation

Five bacterial strains (*Escherichia coli*, *Salmonella enterica*, *Micrococcus luteus*, *Listeria monocytogenes* and *Staphylococcus aureus*) were used for antibacterial testing. The bacterial strains were cultivated on LB agar [LB broth (Lennox), BD Difco^TM^, Washington, WA, USA]. For antibacterial testing, the bacterial cultures were suspended in 100 mL LB broth and grown aerobically for 24 h and continuously shaken at 100 rpm at 37 °C. After 24 h, the bacterial cultures were centrifuged at 10,000 rpm for 10 min. After centrifugation, the supernatant was discarded. The bacteria were washed with 10 mL of 0.9% NaCl solution and centrifuged at 10,000 rpm for 10 min. Then, the bacteria pellets were suspended in a 10 mL of sterile 0.9% NaCl solution. The optical density of each culture was adjusted to 0.07 (in sterile 0.9% NaCl solution) at 600 nm using spectrophotometer (Thermo Scientific NanoDrop^TM^ 2000/2000c Spectrophotometers, Thermo Fisher Scientific, Wilmington, DE, USA).

The bacterial suspension was used to evaluate the control film and gelatin films loaded with different concentrations of RM-WE and RM-MWE for their antibacterial activity by microtiter plate method.

#### 2.6.2. Antibacterial Activity by Microtiter Plate Method

The antibacterial activity of films was determined using 96-well microtiter plates. The control film and gelatin films doped with different concentrations of RM-WE and RM-MWE were surface-sterilized under a UV lamp for 30 min on each side and aseptically cut into discs (5 mm in diameter). The discs were placed in each well of a sterile 96-well microtiter plate. A total of 150 μL of each bacterial suspension was pipetted in microtiter plates. The control wells were prepared with no film (bacterial suspension only) and control film (no RM extract). A blank was maintained by adding 150 μL of 0.9% NaCl solution to the control film and films enriched with RM extracts. The final volume in each well was 150 µL. The plates were placed in an incubator set at 37 °C for 24 h. The test for bacteria and films was performed in four replicates. The absorbance after 24 h incubation was measured using the SpectraMax iD3 microplate reader (Molecular Devices Ltd., San Jose, CA, USA). The plates were mixed on the microplate shaker at medium speed for 4 min allowing uniform distribution of dissolved film and absorbance was read at 600 nm.

### 2.7. Statistical Analysis

The obtained results of film parameters were presented as: mean ± standard deviation (SD). One-way analysis of variance (ANOVA), followed by the Duncan test, was performed to analyze the significant differences between data (*p* < 0.05).

PCA using a covariance matrix was employed in order to compare the mechanical, optical, radical scavenging, and antibacterial properties of the synthesized gelatin-based films. The physicochemical (MC, TS, YM, EAB, L*, a*, b*, opacity), radical scavenging (QUENCHER_DPPH_, QUENCHER_ABTS_), and antimicrobial (against five bacteria strains) properties of the synthesized films were used as active variables in the derivation of the principal components, and the different formulation film samples (containing various amounts of RM-WE and RM-MWE) were projected onto the factor space. The scores and loadings of the data analyzed by PCA were displayed as bi-plot.

Statistical analyses of data were carried out using the Statistica 8.0 software (StatSoft, Tulsa, OK, USA).

## 3. Results and Discussion

### 3.1. Moisture Content in the Prepared Films

The results of MC in gelatin films without and with RM extracts are listed in Table 1.

According to presented data, the MC values of prepared films range between 12.63 and 16.35%. The concentration of RM extracts at the level of 8% the most significantly affected the MC of the synthesized films in comparison with the control film. The film containing 8% of water extract (F+RM-WE-8%) had the highest amount of water (16.35%), whereas the MC of the film incorporating 8% of methanolic extract (F+RM-MWE-8%) was the lowest (12.63%). This observation is rather peculiar and can be the subject of further investigation. The Duncan test indicated that insignificant differences in MC results were observed between control film and films with 4% and 12% of RM extracts (Table 1). Therefore, the MC results suggest that addition of RM extracts did not change radically the hydrophilic characteristic of the obtained films.

Similar MC results (11.8–14.3%) for gelatin films enriched with ethanol-propolis extract and control sample (MC = 13.7%) were reported by Bodini et al. [32]. In contrast, MC in gelatin film incorporated with 9% of aloe gel (23.73%) resulted in a two-fold increase in comparison with the control film (MC = 11.30%) [33].

### 3.2. Mechanical Properties of the Prepared Films

Mechanical parameters are important factors in food packaging materials, which should be controlled due to their connection with tolerance for damage during transport or storage. Incorporation of additional compounds in the polymer matrix result in the alteration of inter-chain interactions and can cause either stiffening or softening of resulting material. These general terms correspond with common mechanical parameters such as tensile strength (TS) and elasticity modulus (YM). Dynamic behavior of polymer film and its ability to sustain the composition under transport condition is well represented by the elongation at break (EAB). In our study the RM extracts containing low molecular weight compounds were introduced to film forming composition. Thus, the molecules of such compounds settled in the polymer matrix. As the data in Table 1 show, the influence of the RM extracts concentration is not straight forward. Addition of 4% RM extracts to the films caused significant differences in the TS values (15.1 and 21.7 MPa for F+RM-WE-4% and F+RM-MWE-4%, respectively) and the effect strongly depends on the nature of solvent. The effect of solvent is opposite to the effect of 8% RM extracts on MC. Moreover, the presence of RM-MWE at the level of 4% in gelatin film led to the highest increase in TS value. However, TS results for gelatin films without antioxidant agent and after incorporation of 8% and 12% RM extracts did not differ significantly (Duncan test, Table 1). Gelatin nanocomposites found in the literature had similar TS values, ranging between 15.5 and 20.8 MPa [34].

**Figure 1:**
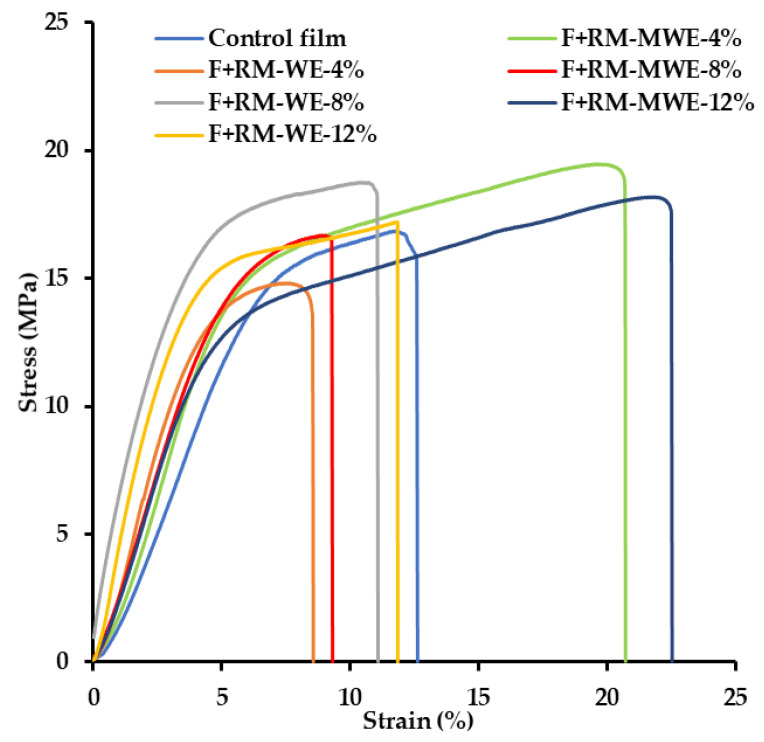
Representative stress–strain curves of the prepared films.

Mechanical properties of all films were measured and the obtained stress–strain curves depicted in Figure 1 were used to determine YM, TS and EAB (Table 1). All the samples showed a typical elastic behavior in the initial region, where the YM was determined. When the yield point was reached, plastic flow started until the film burst.

The YM represents the elastic properties of material, where the higher value indicating less deformation in the material under tensile or compressive stress. It is noteworthy that according to Duncan test the presence of RM extracts in the synthesized films did not change significantly their YM values (310–394 MPa) (Table 1).

For comparison, films prepared from gelatin after addition of different amounts (5–200 g/100 g) of ethanol-propolis extract possessed lower elastic modulus results (8.4–11.3 MPa) [32]. However, similar YM results for gelatin-based films enhanced with nanoclay (386.4 MPa) and silver nanoparticles (303.7 MPa) were observed by Kanmani and Rhim [34]. This indicates that RM extracts show properties more similar to nanoclays or metal nanoparticles than to propolis containing lipid compounds that significantly plastify gelatin.

On the other hand, only the highest concentration of RM-MWE in the prepared film caused a significant increase of the EAB (22%) in comparison with film before addition of bioactive additives (EAB = 15%) (Duncan test, Table 1).

Improvement of the flexibility could be attributed to a reduction of intermolecular forces between protein chains due to the role of RM-MWE as a plasticizer [35]. Furthermore, it can be noted that prepared films generally had low EAB and relatively high stiffness (Table 1). In this context it is crucial to implement additional amounts of plasticizers in further studies.

### 3.3. Color Parameters and Opacity of the Prepared Films

Color parameters and opacity are important measures of the film appearance, which affect consumer acceptance. The values of lightness (L*), redness (a*), yellowness (b*), color difference (ΔE), and opacity of gelatin films regarding the transparency are presented in Table 2.

The Duncan test indicated that the incorporation of RM extracts significantly affected the color of the film surface (*p* < 0.05) by decreasing L* and a* values and increasing b* values. In fact, addition of RM-WE and RM-MWE at 8% concentration reduced film lightness from 91.4 to 90.7 and 90.6, respectively.

It can be noted that the films doped with RM extracts had higher b* values (ranging from −7.4 to −10.1) than control sample (−12.0), turning the films color to more yellow. In contrary, the redness (a*) values was the highest for films without and with the lowest concentration (4%) of RM extracts (Table 2). These variations of films’ color were caused by the natural pigments, mainly chlorophylls, present in RM extracts [36].

The analysis of the films’ opacity revealed that control film was the lightest (0.42 mm^−1^), while the incorporation of RM extracts increased the darkening of gelatin films (opacity = 0.67–0.73 mm^−1^). This can be explained by the fact that natural pigments present in the RM extracts absorb specific wavelengths of visible light, affecting the opacity of doped gelatin films.

Similar opacity results (0.63–1.21 mm^−1^) for gelatin-based films were published for films prepared with papaya peel microparticles described by de Moreaes Crizel et al. [37].

Transparency of the packaging material plays a crucial role in the acceptability of the consumers. It is preferable, when packaged product could be seen directly; therefore, high values of opacity are undesirable.

### 3.4. Microstructure of the Prepared Films

**Figure 2:**
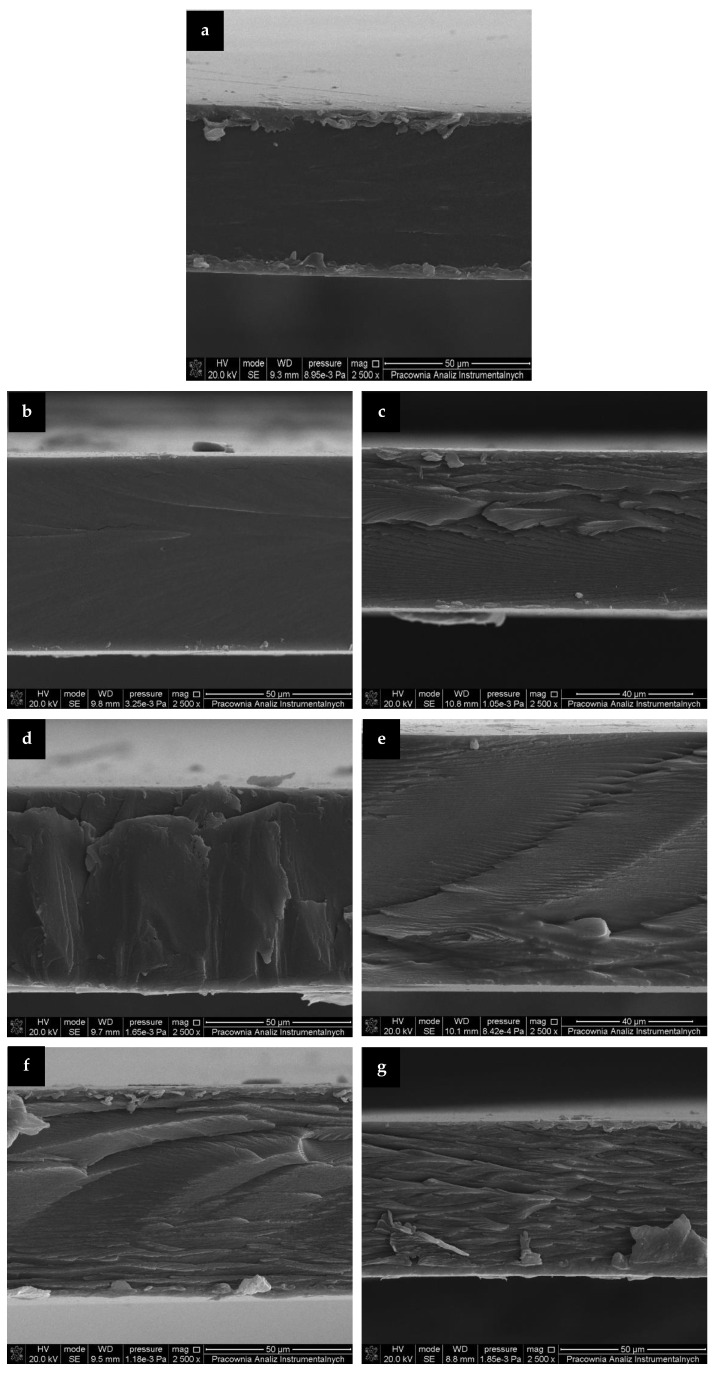
Scanning electron micrographs of cross-sections at 2500× magnification of the prepared gelatin films: (**a**) control film, (**b**) F+RM-WE-4%, (**c**) F+RM-MWE-4%, (**d**) F+RM-WE-8%, (**e**) F+RM-MWE-8%, (**f**) F+RM-WE-12%, and (**g**) F+RM-MWE-12%.

The films’ morphology obtained from the mechanical uniaxial stretching of prepared samples was evaluated by SEM and representative images were presented in Figure 2. As seen, control gelatin film revealed a smooth and homogeneous cross section. The addition of RM extracts, cause the cross sections to became increasingly rough. Inner layers and irregularities in the cross sections of the enhanced films (Figure 2c–g) may be related to increased rigidity and stiffness due to cross linking of gelatin caused by addition of phenolic acids present in RM extracts [18,38]. However, it should be emphasized that SEM images depicted that films containing extracts had uniform structure, without visible pores, which could enhance undesirable permeability of moisture and gases. The homogeneity of the films’ structure was provided by a great compatibility between gelatin and RM extracts due to their hydrophilic nature.

Similar appearance of the microstructure of the rabbit skin gelatin films and gelatin-based films incorporated with catechin–lyzosyme was observed by Ma et al. [39] and Rawdkuen et al. [40], respectively.

### 3.5. Radical Scavenging Activity of Rapeseed Meal Extracts and the Prepared Films

**Figure 3:**
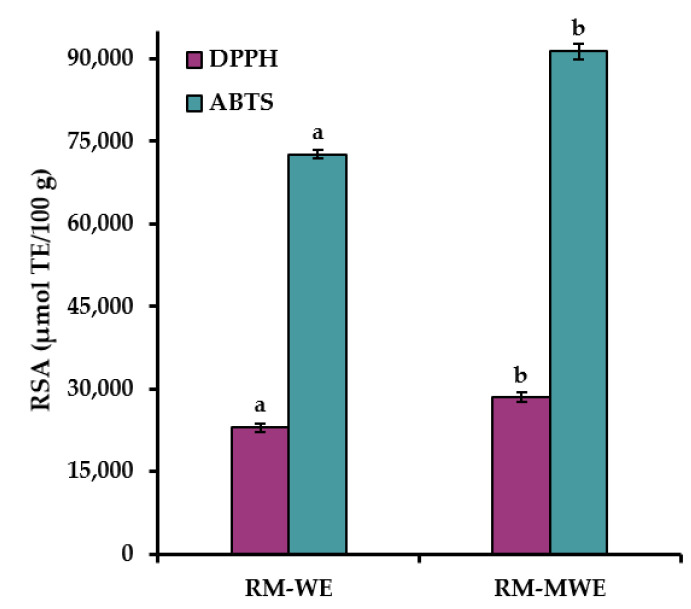
Radical scavenging activity of RM extracts determined by DPPH and ABTS methods. Bars with different letters (a, b) represent statistical differences (one-way ANOVA and Duncan test, *p* < 0.05) between radical scavenging activity of RM-WE and RM-MWE.

The RSA results of RM extracts determined by the commonly used DPPH and ABTS methods are presented in Figure 3.

It can be noted that ABTS values for RM extracts were approximately three times higher than DPPH results. This can be explained by the fact, that ABTS radical cation is reactive towards most antioxidants including both hydrophilic and lipophilic compounds, whereas DPPH radical can only be dissolved in organic media, especially in alcoholic media, which is an important limitation for determination of hydrophilic antioxidants. Moreover, water extracts of RM had significantly lower RSA analyzed by two spectrophotometric assays (DPPH = 22,964 μmol TE/100 g and ABTS = 72,665 μmol TE/100 g) in comparison with DPPH (28,474 μmol TE/100 g) and ABTS (91,351 μmol TE/100 g) for RM-MWE (Duncan test, Figure 3). This suggests that the methanol–water mixture (1:1 *v*/*v*) was a more efficient solvent than water for extraction of antioxidants from RM sample.

The obtained DPPH results for RM-WE and RM-MWE are in good agreement with those presented in our previous reports (DPPH = 13,620–19,930 μmol/100 g and 21,320–25,070 μmol/100 g for aqueous and 50% methanolic extracts of RM samples, respectively) [41,42].

Therefore, a high RSA results of RM extracts indicate that antioxidants present in RM-WE and RM-MWE can be applied for the production of active films with potent antioxidant properties.

Antioxidant packaging is one of the major categories of active packaging protecting contained food from the oxidative degradation. The radical scavenging properties of the films were evaluated by spectrophotometric QUENCHER_DPPH_ and QUENCHER_ABTS_ assays based on direct contact of DPPH radical and ABTS radical cation, respectively with investigated film samples. Therefore, non-extractable compounds with antioxidant potential present in the synthesized films can be determined by these analytical procedures.

**Figure 4:**
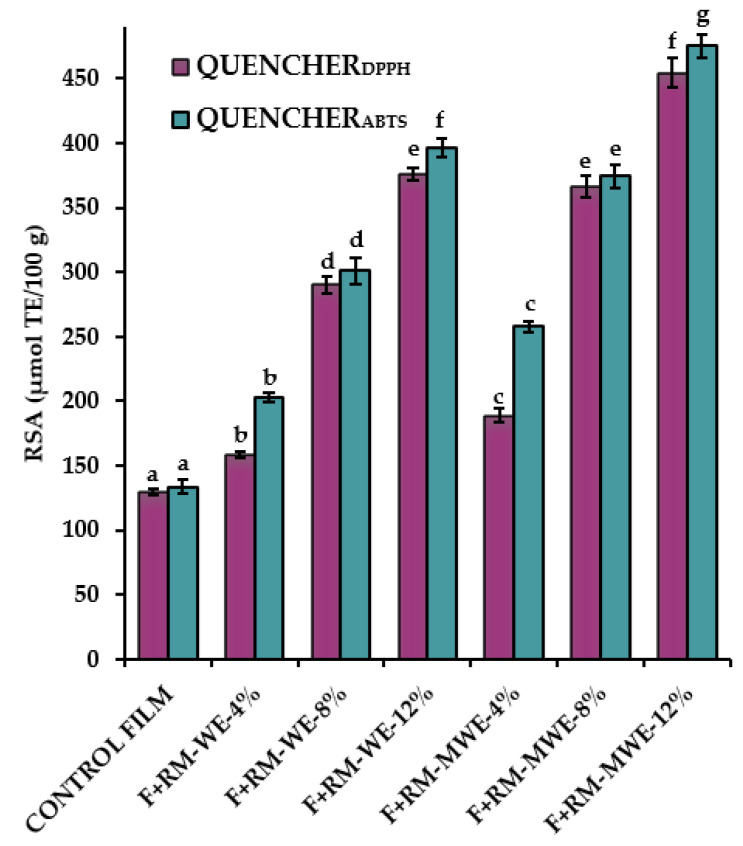
Radical scavenging properties of the prepared films determined by QUENCHER_DPPH_ and QUENCHER_ABTS_ methods. Bars with different letters (a–g) represent statistical differences (one-way ANOVA and Duncan test, *p* < 0.05) between radical scavenging activity of gelatin films with RM-WE and RM-MWE.

As seen in the Figure 4, the QUENCHER_DPPH_ and QUENCHER_ABTS_ values of films increased with increasing the extracts concentration.

The control film also presented relatively high radical scavenging properties (QUENCHER_DPPH_ = 129.42 μmol TE/100 g and QUENCHER_ABTS_ = 133.49 μmol TE/100 g).

For comparison, gelatin-based film without additives had high RSA = 53% measured by classical DPPH method [11].

The Duncan test indicated that RSA values for gelatin films with RM-WE and RM-MWE were significantly higher than the control film (without RM extracts). Incorporation of 12% RM-WE and RM-MWE resulted in the highest QUENRCHER_DPPH_ (376.21 and 454.53 μmol TE/100 g, respectively) and QUENRCHER_ABTS_ (396.40 and 475.60 μmol TE/100 g, respectively). It is noteworthy that RSA of the studied films analyzed by QUENRCHER_ABTS_ method exhibited higher values (133.49–475.60 μmol TE/100 g) in comparison with those measured by QUENCHER_DPPH_ assay (129.42–454.53 μmol TE/100 g). In general, gelatin films incorporating RM-MWE had higher radical scavenging properties in comparison with those doped with RM-WE.

The significant increase of the DPPH (5.1–76.2%) and ABTS (12.9–88.1%) of gelatin films after addition of curcumin at various concentrations (0–1.5%) measured by direct contact with DPPH radical and ABTS radical cation, respectively, was observed by Roy and Rhim [43].

Radical scavenging properties of the cast gelatin films enriched with RM extracts can be an interesting and simple alternative to synthetic polymer films in protection of packaged food against undesirable processes such as lipids oxidation.

### 3.6. Antimicrobial Properties of the Prepared Films

**Figure 5:**
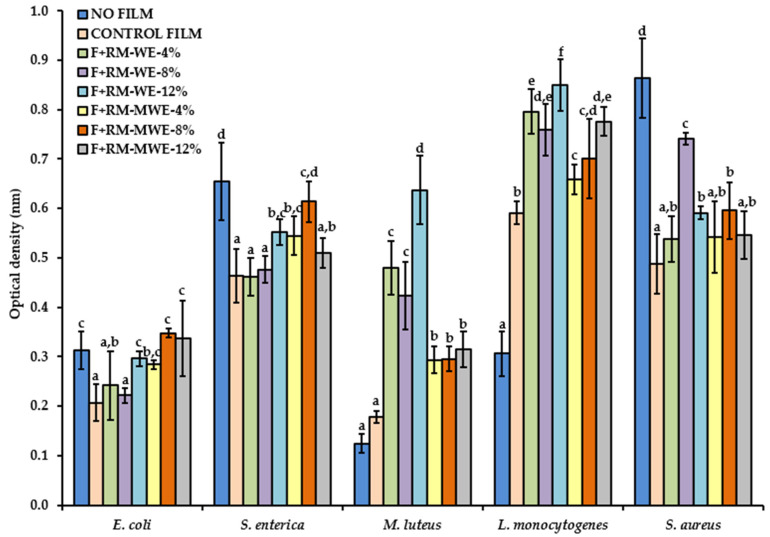
Antimicrobial activity of gelatin films enriched with different concentrations of RM-WE and RM-MWE against Gram-negative bacteria (*E. coli* and *S. enterica*) and Gram-positive bacteria (*M. luteus*, *L. monocytogenes*, and *S. aureus*). Bars with different letters (a–f) represent statistical differences (one-way ANOVA and Duncan test, *p* < 0.05) between the gelatin films with RM-WE and RM-MWE for each of the bacteria.

The antibacterial activity of the gelatin films enriched with 4, 8 and 12% of RM-WE as well as 4, 8 and 12% of RM-MWE displayed activity against the test pathogens. A similar trend of microbial inhibition was observed based on the Gram-staining character of the strains (Figure 5).

The Gram-negative bacteria *E. coli* showed significant inhibition with control film, F+RM-WE-4%, F+RM-WE-8% and F+RM-MWE-4% but not with the films F+RM-WE-12%, F+RM-MWE-8% and F+RM-MWE-12% as compared to the variant of the experiment with no film. While *S. enterica* displayed inhibition with control film, F+RM-WE-4%, F+RM-WE-8%, F+RM-WE-12%, F+RM-MWE-4% and F+RM-MWE-12% but not with F+RM-MWE-8%. Conversely, the Gram-positive bacterial strains (*M. luteus* and *L. monocytogenes*) showed that the wells with films caused no inhibition when compared to the variant of the experiment with no film. The films with higher concentrations of extracts displayed higher Gram-positive bacterial activity, in the case of films F+RM-WE-4%, F+RM-WE-8% and F+RM-WE-12% for both the strains. Moreover, *S. aureus* (Gram-positive) displayed a pattern similar to Gram-negative bacteria with significant inhibition observed for all the tested films.

Overall, the RM extracts were more active against Gram-negative bacteria than against Gram-positive bacteria. The most susceptible bacteria were *S. enterica* followed by *E. coli*, while the most resistant bacteria were *L. monocytogenes*, followed by *M. luteus* with the exception of *S. aureus* which was found to be susceptible. Among all the tested, the highest antibacterial activity was recorded with the control film, F+RM-WE-4% and F+RM-WE-8%.

### 3.7. Principal Component Analysis

**Figure 6:**
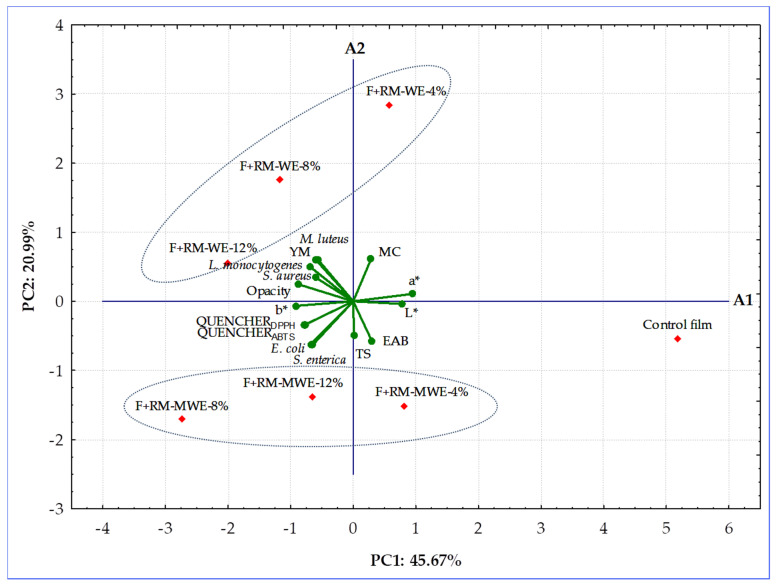
Biplot of scores and loadings of data obtained from mechanical, optical, radical scavenging, and antibacterial properties of the prepared gelatin-based films. Blue circles indicate the distribution of variables in two groups based on the activity of the gelatin films with RM-WE against Gram-positive bacteria and films with RM-MWE against Gram-negative bacteria.

The synthesized gelatin-based films without and with RM extracts were classified according to their physicochemical (MC, TS, YM, EAB, L*, a*, b*, opacity), radical scavenging (QUENCHER_DPPH_, QUENCHER_ABTS_), and antimicrobial (against five bacteria strains: *E. coli*, *S. enterica*, *M. luteus*, *L. monocytogenes,* and *S. aureus*) properties employing PCA. This chemometric tool was applied to observe any possible groups within the prepared gelatin films. The first two principal components took 66.66% (PC1 = 45.67% and PC2 = 20.99%, respectively) of the total variation into account. The distribution of the most significant variables along the two first principal components and the groupings and/or the differences among fortified gelatin films are presented in a bi-plot (Figure 6).

The PCA graph revealed that the control film and two films doped with the lowest content (4%) of RM extracts having the highest redness (a* values) and antibacterial activity as well as the lowest yellowness (b* values), and RSA were located to the right in the score plot and had positive values for PC1. However, four enriched films more yellowness b* values with high radical scavenging and low antimicrobial properties were situated at the left in the diagram and had negative values for PC1 (Figure 6). The films with 8% and 12% of RM water extracts (F+RM-WE-8% and F+RM-WE-12%) characterized by inactivity against *M. luteus* and *L. monocytogenes*, having the same EAB values, high opacity, moderate RSA values, and low redness a* values created evidently distinct cluster. However, the highest results of QUENCHER_DPPH_, QUENCHER_ABTS_ and inactivity against the two Gram-negative bacterial strains (*E. coli* and *S. enterica*) moved films fortified with 8 and 12% of RM methanolic extracts (F+RM-MWE-8% and F+RM-MWE-12%) to the third quadrant of PCA graph (Figure 4, Figure 5 and Figure 6, Table 1 and Table 2). Furthermore, gelatin films before and after addition of RM methanolic extracts with lower MC and YM values were located under the A1 axis. The control film with the longest distance from other film samples revealed the lowest YM, yellowness (b* value), opacity and RSA results, as well as the most lightness (L*) and redness (a*), and high antibacterial activity. It can be noted that in the groups of studied films, RM-WE and RM-MWE added at final concentrations of 8% and 12% were the most selective RM amounts, since a closer association of their antibacterial activity with tested bacteria in comparison with films containing 4% of RM-WE and RM-MWE extracts was observed. As seen, MC and redness (a* values) of films had positive loadings on the PC1 and PC2, while YM, opacity and three Gram positive bacteria strains (*M. luteus*, *L. monocytogenes*, and *S. aureus*) were the variables with negative loadings on PC1 and positive loadings on PC2. However, yellowness (b* values), RSA and two Gram-negative bacterial strains (*E. coli* and *S. enterica*) revealed loadings on the negative dimension of both PC1 and PC2, whereas TS, EAB and lightness (L* values) were the features with positive loadings on PC1 and negative on PC2 (Figure 6).

Additionally, YM of the discussed films was significantly positively correlated to the opacity (r = 0.8639, *p* = 0.0122) and antibacterial activity against *L. monocytogenes* (r = 0.7700, *p* = 0.0429), while antibacterial activity of synthesized films against *L. monocytogenes* positively associated to their opacity (r = 0.7871, *p* = 0.0357) and antibacterial activity against *M. luteus* (r = 0.9034, *p* = 0.00529). Moreover, there were high positive correlations between QUENCHER_DPPH_, and QUENCHER_ABTS_ results (r = 0.9828, *p* = 0.000074) as well as QUENCHER_DPPH_ and antibacterial activity against *E. coli* (r = 0.7780, *p* = 0.0394). Additionally, the calculated r values suggest that there were significant (*p* = 0.00835–0.0478), positive associations among films’ parameters such as QUENCHER_ABTS_—*E. coli* (r = 0.8265), yellowness (b* values)—*S. aureus* (r = 0.7591), *E. coli*—*S. enterica* (r = 0.8233), and lightness (L* values)—redness (a* values) (r = 0.8835). In contrast, a significant (*p* = 0.000081–0.0398), negative correlations for redness (a* values) and yellowness (b* values) (r = −0.9821), lightness (L* values)—yellowness (b* values) (r = −0.9534), lightness (L* values)—*S. aureus* (r = −0.7772), and MC—*S. enterica* (r = −0.8577) were found.

The results obtained by PCA indicated that the films can be clearly distinguished by composition and properties, indication that type of RM extracts and their concentrations added to films caused differences among themselves. PCA allowed to verify that the gelatin-based films fortified with RM-MWE having good mechanical, optical and radical scavenging properties would be the best choice for protection of packaged food against Gram negative bacteria and oxidative degeneration, whereas packaging materials containing RM-WE were more effective against Gram-positive bacteria.

## 4. Conclusions

The RM, as a by-product of rapeseed oil industry rich in antioxidant compounds was successfully incorporated into gelatin films. Addition of water and methanolic extracts of RM in the concentrations range between 4 and 12% did not cause significant changes in the elastic modulus of the synthesized films, whereas films’ color and opacity were clearly affected by RM-WE and RM-MWE supplementation. Interestingly, the enhancement of films’ flexibility was detected after addition of RM extracts, which may be related to the films microstructures and interactions between components present in RM and protein chains of gelatin. Moreover, with the increasing amount of RM extracts incorporated in the gelatin-based films, their higher radical scavenging properties were achieved. The enriched gelatin films were more active against Gram-negative bacteria (specifically *S. enterica*) than against Gram-positive bacteria (*L. monocytogenes*). By using PCA, it was possible to visualize, by the separation of films in clusters, the presence of the various RM extracts at different concentrations in the prepared films.

Further research is aimed at characterizing the composition of RM extracts for relevant compounds as well as carry out tests on real food systems for effective active food packaging applications.

## Figures and Tables

**Table 1:** Moisture content and mechanical properties of the prepared films.

Film Type	MC * ± SD (%)	TS * ± SD (MPa)	YM * ± SD (MPa)	EAB * ± SD (%)
Control film	14.71 ± 0.38 ^c^	17.3 ± 0.1 ^b^	245 ± 32 ^a^	15 ± 3 ^a,b^
F+RM-WE-4%	15.15 ± 0.31 ^c^	15.1 ± 0.3 ^a^	394 ± 32 ^b^	9 ± 1 ^a^
F+RM-WE-8%	16.35 ± 0.31 ^d^	18.8 ± 0.6 ^b^	326 ± 60 ^a,b^	11 ± 3 ^a^
F+RM-WE-12%	13.88 ± 0.22 ^b^	17.5 ± 1.0 ^b^	336 ± 116 ^a,b^	11 ± 2 ^a^
F+RM-MWE-4%	14.28 ± 0.32 ^b,c^	21.7 ± 2.4 ^c^	310 ± 15 ^a,b^	19 ± 6 ^b,c^
F+RM-MWE-8%	12.63 ± 0.39 ^a^	17.1 ± 0.8 ^b^	325 ± 63 ^a,b^	9 ± 1 ^a^
F+RM-MWE-12%	14.98 ± 0.43 ^c^	17.9 ± 4.0 ^b^	319 ± 40 ^a,b^	22 ± 4 ^c^

* *n* = 3; SD—standard deviation; different letters ^(a–d)^ within the same column indicate significant differences between moisture content and mechanical properties of the studied films (one-way ANOVA and Duncan test, *p* < 0.05).

**Table 2:** Color and opacity properties of the prepared films.

Film Type	L* ± SD	a* ± SD	b* ± SD	ΔE* ± SD	Opacity* (mm^−1^)
Control film	91.4 ± 0.1 ^d^	1.1 ± 0.1 ^f^	−12.0 ± 0.1 ^a^	-	0.42 ± 0.01 ^a^
F+RM-WE−4%	91.1 ± 0.1 ^b,c^	0.8 ± 0.1 ^e^	−10.1 ± 0.2 ^b^	1.9 ± 0.3 ^a^	0.73 ± 0.01 ^c,d^
F+RM-WE-8%	90.7 ± 0.1 ^a^	0.3 ± 0.1 ^b^	−7.9 ± 0.3 ^d^	4.3 ± 0.3 ^c^	0.68 ± 0.02 ^b^
F+RM-WE-12%	91.1 ± 0.2 ^b,c^	0.5 ± 0.1 ^c^	−9.1 ± 0.7 ^c^	2.9 ± 0.7 ^b^	0.73 ± 0.01 ^d^
F+RM-MWE-4%	91.0 ± 0.1 ^b^	0.7 ± 0.1 ^d^	−9.4 ± 0.1 ^c^	2.6 ± 0.1 ^b^	0.67 ± 0.01 ^b^
F+RM-MWE-8%	90.6 ± 0.1 ^a^	0.2 ± 0.1 ^a^	−7.4 ± 0.2 ^e^	4.7 ± 0.2 ^c^	0.71 ± 0.02 _c_
F+RM-MWE-12%	91.2 ± 0.1 ^c^	0.6 ± 0.1 ^c^	−9.5 ± 0.5 ^c^	2.5 ± 0.5 ^b^	0.67 ± 0.02 ^b^

* n = 5; SD—standard deviation; different letters ^(a–f)^ within the same column indicate significant differences between color parameters and opacity of the studied films (one-way ANOVA and Duncan test, *p* < 0.05).

## Data Availability

The data presented in this study are available on request from the corresponding author.
